# Combining Congenital Heart Surgical and Interventional Cardiology Outcome Data in a Single Database: The Development of a Patient-Centered Collaboration of the European Congenital Heart Surgeons Association (ECHSA) and the Association for European Paediatric and Congenital Cardiology (AEPC)

**DOI:** 10.1177/21501351231168829

**Published:** 2023-07-06

**Authors:** Jeffrey P Jacobs, Thomas Krasemann, Claudia Herbst, Zdzislaw Tobota, Bohdan Maruszewski, Jose Fragata, Tjark Ebels, Vladimiro L Vida, Ilkka Mattila, Andrzej Kansy, Boulos Asfour, Jürgen Hörer, Attilio A Lotto, M Sertaç Çiçek, Petru Liuba, Sven Dittrich, Massimo Chessa, Regina Bökenkamp, Gurleen Sharland, Katarina Hanséus, Nico A Blom, George E Sarris

**Affiliations:** 1 Congenital Heart Center, Division of Cardiovascular Surgery, Departments of Surgery and Pediatrics, University of Florida, Gainesville, FL, United States of America; 2 Department of Paediatric Cardiology, Sophia Children's Hospital, Rotterdam, The Netherlands; 3 Landesklinikum Baden-Mödling, Mödling, Austria; 4 Pediatric Cardiothoracic Surgery, Children's Memorial Health Institute, Warsaw, Poland; 5 Hospital de Santa Marta, NOVA Medical School, Lisbon, Portugal; 6 Department of Cardiothoracic Surgery, University Medical Center Groningen, Groningen, The Netherlands; 7 Pediatric and Congenital Cardiac Surgery Unit, Department of Cardiac, Thoracic, Vascular Sciences and Public Health, University of Padua, Padua, Italy; 8 Department of Pediatric Cardiac Surgery, Hospital for Children and Adolescents, 3835 University of Helsinki, Helsinki, Finland; 9 Department of Pediatric Cardiac Surgery, Pediatric Heart Center, University Hospital Bonn (UKB), Bonn, Germany; 10 Department of Congenital and Pediatric Heart Surgery, German Heart Center Munich, Munich, Germany; 11 Division of Congenital and Pediatric Heart Surgery, University Hospital of Munich, Ludwig-Maximilians-Universität, Munich, Germany; 12 Pediatric Cardiac Surgery, Alder Hey Children's Hospital, Liverpool, UK; 13 Istanbul University Faculty of Medicine, Department of Cardiovascular Surgery, Istanbul, Turkey; 14 Department of Cardiology, Pediatric Heart Center, Skåne University Hospital, Lund, Skåne, Sweden; 15 5193 Lund University, Lund, Skåne, Sweden; 16 Department of Pediatric Cardiology, Friedrich-Alexander-Universitat Erlangen-Nurnberg, Erlangen, Germany; 17 ACHD Unit, Department of Pediatric and Adult Congenital Disease, IRCCS Policlinico San Donato, San Donato Milanese, Italy; 18 Vita Salute San Raffaele University, Milan, Italy; 19 Department of Pediatric Cardiology, Leiden University Medical Center, Leiden, The Netherlands; 20 Department of Congenital Heart Disease, Evelina London Children's Hospital, Guy's and St Thomas’ NHS Foundation Trust, London, United Kingdom; 21 Department of Paediatric Cardiology, Skåne University Hospital, Lund, Sweden; 22 Department of Clinical Sciences, 5193 Lund University, Lund, Sweden; 23 Paediatric Cardiology, Amsterdam University Medical Center, Amsterdam, The Netherlands; 24 Athens Heart Surgery Institute, Athens, Greece

**Keywords:** nomenclature, database, pediatric, congenital heart disease, pediatric heart disease, interventional cardiology, cardiac surgery

## Abstract

The European Congenital Heart Surgeons Association (ECHSA) Congenital Database (CD) is the second largest clinical pediatric and congenital cardiac surgical database in the world and the largest in Europe, where various smaller national or regional databases exist. Despite the dramatic increase in interventional cardiology procedures over recent years, only scattered national or regional databases of such procedures exist in Europe. Most importantly, no congenital cardiac database exists in the world that seamlessly combines both surgical and interventional cardiology data on an international level; therefore, the outcomes of surgical and interventional procedures performed on the same or similar patients cannot easily be tracked, assessed, and analyzed. In order to fill this important gap in our capability to gather and analyze information on our common patients, ECHSA and The Association for European Paediatric and Congenital Cardiology (AEPC) have embarked on a collaborative effort to expand the ECHSA-CD with a new module designed to capture data about interventional cardiology procedures. The purpose of this manuscript is to describe the concept, the structure, and the function of the new *AEPC Interventional Cardiology Part of the ECHSA-CD*, as well as the potentially valuable synergies provided by the shared interventional and surgical analyses of outcomes of patients. The new *AEPC Interventional Cardiology Part of the ECHSA-CD* will allow centers to have access to robust surgical and transcatheter outcome data from their own center, as well as robust national and international aggregate outcome data for benchmarking. Each contributing center or department will have access to their own data, as well as aggregate data from the *AEPC Interventional Cardiology Part of the ECHSA-CD*. The new *AEPC Interventional Cardiology Part of the ECHSA-CD* will allow cardiology centers to have access to aggregate cardiology data, just as surgical centers already have access to aggregate surgical data. Comparison of surgical and catheter interventional outcomes could potentially strengthen decision processes. A study of the wealth of information collected in the database could potentially also contribute toward improved early and late survival, as well as enhanced quality of life of patients with pediatric and/or congenital heart disease treated with surgery and interventional cardiac catheterization across Europe and the world.

## Introduction

It is well recognized that optimal care of patients with congenital heart disease (CHD) requires a multidisciplinary approach centered around the needs of the patient. Management of the patient may involve various interventional cardiology procedures, surgical operations, or even combined hybrid procedures, and frequently more than once during the life of the patient. Outcomes depend on multiple factors including the complexity of the disease itself, other patient-related factors including concomitant pathologies and comorbidities, the clinical status of the patient, and factors external to the patient that are related to the available resources and organization of the health care team. The complex interactions of all these features render the evaluation of outcomes sometimes challenging. It is also clear that the determination of outcomes increasingly should involve not only tracking mortality but also, perhaps most importantly, various complications (many of which are of a general nature, while others are procedure-specific), and other quality metrics.

Efforts to evaluate the benefit to our patients of operations and transcatheter procedures depend on collecting the relevant information in well-organized databases with a high degree of participation and coverage, a task which requires the existence of a common nomenclature to be used by all data contributors^1–7^ and the development of appropriate analytical tools.^8–13^ The European Congenital Heart Surgeons Association (ECHSA) Congenital Database (CD) is the second largest clinical pediatric and congenital cardiac surgical database in the world and the largest in Europe, where various smaller national or regional databases exist. ECHSA-CD has also recently developed powerful artificial intelligence and machine learning–based methodologies that will enhance the art and science of pediatric and congenital cardiac outcomes analysis.^12,13^ On the other hand, despite the remarkable increase in interventional cardiology procedures over recent years, only scattered national or regional databases of such procedures exist in Europe. To date, few national databases combine both cardiac surgical and interventional cardiology data in the same database.^14,15^ Most importantly, however, no congenital cardiac database exists in the world that seamlessly combines both surgical and interventional cardiology data on an international level; therefore, the outcomes of surgical and interventional procedures performed on the same or similar patients cannot easily be tracked, assessed, and analyzed. In order to fill this important gap in our capability to gather and analyze information on our common patients, ECHSA and The Association for European Paediatric and Congenital Cardiology (AEPC) have embarked on a collaborative effort to expand the ECHSA-CD with a new module designed to capture data about interventional cardiology procedures. The purpose of this manuscript is to describe the concept, the structure, and the function of the new *AEPC Interventional Cardiology Part of the ECHSA-CD*, as well as the potentially valuable synergies provided by the shared interventional and surgical analyses of outcomes of patients.

## Methods—History of the Project

The AEPC (https://www.aepc.org/) was founded in 1963. Currently, more than 1,200 members are organized in a network of specialists who are committed to the practice and advancement of Congenital Cardiology and closely related fields. The AEPC members originate from 32 European countries, but there are several members from outside Europe, too. The mission of AEPC is (https://www.aepc.org/our-mission):

- “(a) Knowledge of the normal and diseased heart and circulation in a growing individual and
- (b) Exchange of expertise between experts from Europe and globally and
- (c) Continuous medical education
- (d) Harmonizing training in Paediatric Cardiology and its subspecialties in Europe. This is done by means of creating European recommendations for training and by organizing several Teaching Courses for Fellows in training.”

Several working groups represent the different aspects of diagnosis and treatment of congenital cardiac patients from fetal life to geriatric age. The 14 working groups of AEPC are responsible for the development of education, training, and exchange of knowledge within the different subspecialties. One important subspecialty is organized in the Interventional Working Group, where current knowledge and new developments are frequently shared. Apart from pediatric and adult cardiologists, several cardiac surgeons are also members. The interdisciplinary collaboration is also reflected in the relationship with other organizations focusing on the care of patients with congenital heart disease.

**Figure 1.**
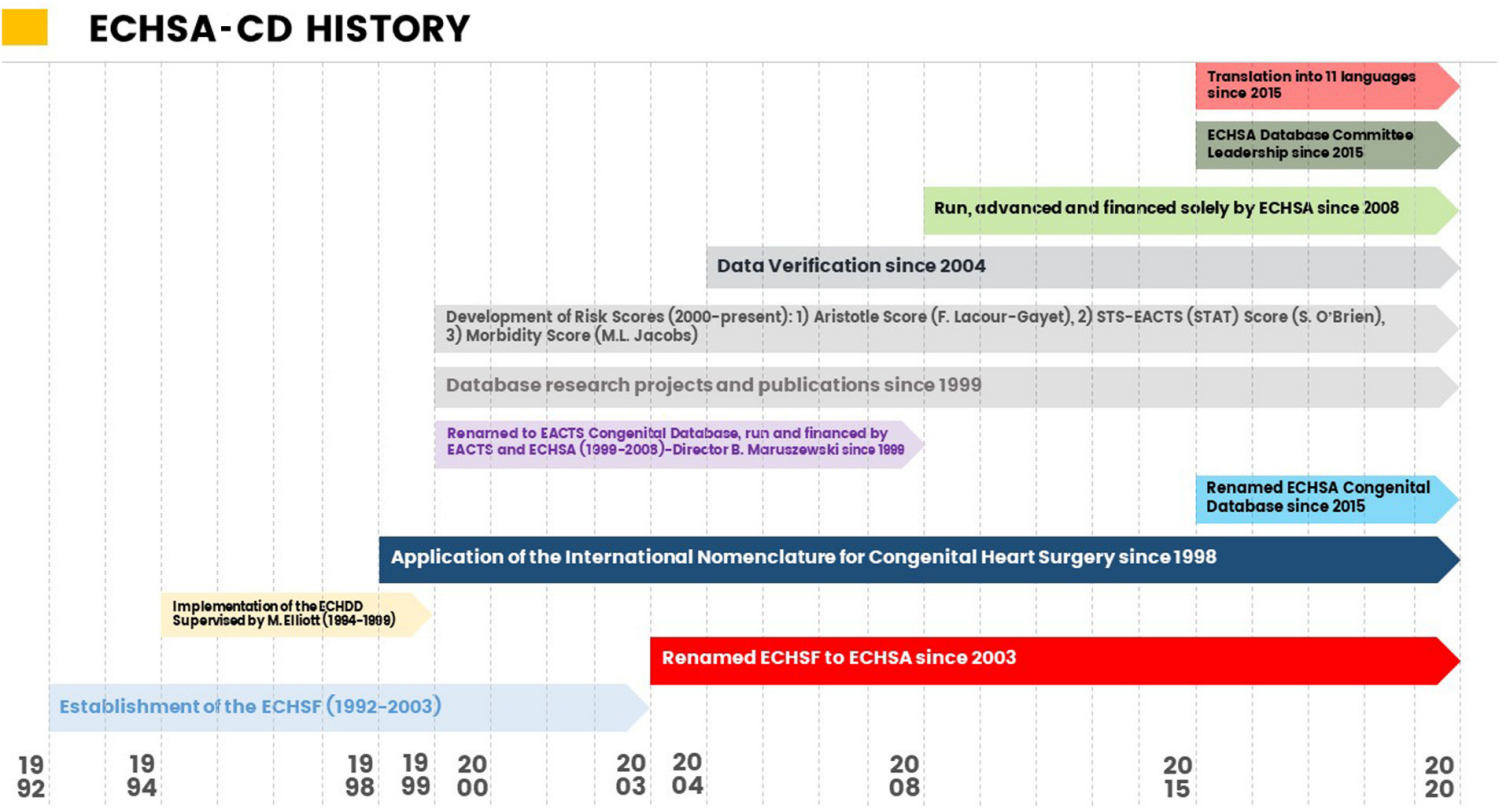
History of ECHSA-CD. Abbreviations: EACTS, European Association for Cardio-Thoracic Surgery; ECHDD, European Congenital Heart Defects Database; ECHSA-CD, European Congenital Heart Surgeons Association Congenital Database; ECHSF, European Congenital Heart Surgeons Foundation.

The ECHSA (https://www.echsa.org/) arose in 2003 following the renaming of its parent society, the European Congenital Heart Surgeons Foundation, which had been established in 1992. The development of the congenital cardiac surgical database began in 1994. ECHSA-CD was initially named the European Congenital Heart Defects Database, renamed as the European Association for Cardio-Thoracic Surgery Congenital Database (EACTS CD) in 1999, and acquired its final name of “ECHSA Congenital Database” in 2015, owned and directed by ECHSA. Figure 1 documents the history of ECHSA-CD.

Over the years, a strong collaboration and harmonization with The Society of Thoracic Surgeons (STS) Congenital Heart Surgery Database (CHSD) was maintained by using common nomenclature and common data structure and fields.^1,2,3,8^ For standardization, the International Pediatric and Congenital Cardiac Code (IPCCC)^1,2,3^ is used for coding. The translation into various languages enables further integration of the international community of pediatric and congenital cardiac care. The ECHSA-CD is based in Europe as a worldwide database, and ECHSA-CD is open to everyone. By May 2023, data pertaining to 303,892 patients and 358,052 operations have been collected. The database functions allow users to create customized online reports on subgroups of patients and procedures. Verification of the completeness and accuracy of the data in ECHSA-CD is performed utilizing “*source data verification*.”^16–18^ The technical details of “*source data verification*” have been previously published^16–18^ and include an audit of the data at individual hospitals (for both completeness and accuracy^19^), with comparison of the data in ECHSA-CD to the primary source of the data at the hospital (eg, hospital operative logs and hospital medical records). (This process of “*source data verification*” that is currently applied to ECHSA-CD will continue to be applied in ECHSA-CD and will also be applied to the *AEPC* *Interventional Cardiology Part of the ECHSA-CD*.) The aims of collecting data with ECHSA-CD across Europe on the outcomes of congenital cardiac surgery procedures are multifactorial and include:

- measure and assess quality;
- provide a platform for benchmarking individual and programmatic results in comparison to national and international aggregate data (in the domains of mortality and morbidity);
- determine risk factors;
- improve quality;
- generate new knowledge, in other words, research; and
- enable predictive statistical analysis according to pathologies and procedures from various centers and countries, helping to define official European standards available for the scientific community and health care.

The research publications of ECHSA-CD are summarized on the official website of ECHSA (https://echsacongenitaldb.org/). For transcatheter interventions in patients with CHD, several local and national databases exist. These local and national databases differ markedly, even if they all have the same goals of quality assessment and quality improvement. In some countries, only a minimal dataset (eg, the age of the patient at the procedure and the type of procedure) is collected, while in others, the depth of data collection is remarkable. Many local databases are programmed by single specialists in information technology, and not all of these databases can be considered user-friendly. The reports which can be obtained from these databases differ. While some allow benchmarking of outcomes (ie, National Institute for Cardiovascular Outcomes Research [NICOR] in the United Kingdom [https://www.nicor.org.uk/]), others allow pure counting of procedures, sometimes with, and sometimes without, tracking specified complications. One of these national databases allows a comparison of key quality indicators and key procedural performance indicators about transcatheter interventions with cardiac surgical data.^15^ Importantly, none of these databases allows direct comparison of data on an international level about transcatheter interventions with cardiac surgical data, which is especially valuable for diseases, that are treatable both by transcatheter and surgical intervention.

For several years, the AEPC Interventional Working Group had plans to develop a European database with the following criteria:

- Data can be entered in a user-friendly manner
- The database will allow both the entry of basic data alone and the entry of in-depth data, according to the needs of each single center or the specific national requirements for quality control.
- The database will generate reports of the outcomes of specified procedures.
- The database will generate reports of own center data, as well as reports of national and Europe-wide data.

The ECHSA-CD had also long desired to include data about interventional cardiology in their analyses, and several early preliminary discussions of cooperation with the AEPC had taken place. By 2015, cooperation of ECHSA and AEPC had matured, with the ECHSA Secretary General George E. Sarris, MD representing the surgical community in the AEPC Council. ECHSA, under the leadership of Jose Fragata, proposed collaboration on development of an *AEPC Interventional Cardiology Part of the ECHSA-CD*, in the context of the existing ECHSA-CD, and the AEPC, under the leadership of Gurleen Sharland, officially accepted.

## Methods—Rationale of the Project

Development of an *AEPC Interventional Cardiology Part of the ECHSA-CD* in the context of the existing ECHSA-CD has multiple potential advantages:

- The legalities of data protection according to different national law have already been addressed and solved by ECHSA.
- The process of data verification is already established.
- A large body of surgical data already exists in ECHSA-CD, harmonized with the data in STS CHSD.
- Refined data assessment tools have been developed and are available.
- Multiple extant scientific publications demonstrate the scientific power of ECHSA-CD.
- The collaboration between AEPC and ECHSA and the addition of data about interventional cardiology to ECHSA-CD creates the only congenital cardiac database in the world with combined, detailed data about congenital interventional cardiology and congenital cardiac surgery.
- The *AEPC Interventional Cardiology Part of the ECHSA-CD* will create important and unique opportunities for post-market surveillance of implanted devices, which will be especially useful with new European Union initiatives and regulations related to post-market surveillance of medical devices.
- The addition of an *AEPC Interventional Cardiology Part of the ECHSA-CD* to the ECHSA-CD represents a European database collaborative effort supported by the two major and well-established European scientific associations (AEPC and ECHSA) working on quality improvement for the treatment of our common patients with CHD.
- Since a large amount of surgical data spanning more than two decades is already available in ECHSA-CD, outcome assessment, benchmarking, and quality assurance programs will be facilitated.

To realize the agreed collaborative goal, an AEPC representative (TK) was selected by the AEPC Council and appointed by ECHSA as a member of the ECHSA Database Committee, as a liaison with the AEPC Interventional Working Group, with the following objectives:

- to define the specific goals of the project,
- to select and define the data fields to be collected,
- to select and define the outcomes to be tracked, and
- to design the implementation steps.

## Results—Structure and Operation of the Project

In 2019, the first meetings took place involving the ECHSA Database Committee with the new AEPC representative. During these initial meetings, the following objectives were completed and the following decisions were made:

- The needs of the new *AEPC Interventional Cardiology Part of the ECHSA-CD* were established.
- Mandatory and optional demographic data were identified.
- The decision was made to use IPCCC nomenclature for all diagnoses.
- Potential interventional treatments to monitor were considered.
- The decision was made to assure appropriate linkage between diagnosis and the corresponding potential intervention.
- Possible complications associated with these diseases or their associated specific interventions were defined. These complications may cause a deviation from the desired course or may be associated with suboptimal outcome.^20^
- Procedure-related data such as radiation dose and time of exposure will be collected.
- Both interventional cardiac catheterizations and diagnostic cardiac catheterizations will be recorded.
- Each component procedure of multicomponent interventions will be entered into the new *AEPC Interventional Cardiology Part of the ECHSA-CD*.
- Outcome data will consist of intervention success, related morbidity, and mortality.
- Mortality will continue to be defined in all parts of ECHSA-CD, including the new *AEPC Interventional Cardiology Part of the ECHSA-CD*, as Operative Mortality, using the standard definition of Operative Mortality currently used in ECHSA-CD and STS CHSD.^21,22^
- In ECHSA-CD, postoperative length of stay is currently calculated as the amount of time between the completion of the operation and discharge from the hospital. In the new *AEPC Interventional Cardiology Part of the ECHSA-CD*, postprocedural length of stay will be calculated as the amount of time between the completion of the interventional procedure and discharge from the hospital.
- Follow-up data can be added. After 30 days and 90 days post-intervention, the interventional team is reminded by a pop-up window to enter these follow-up data.
- The Appendix provides a Quick Users’ Guide that includes multiple screen captures of the user-friendly data entry interface that was developed for the *AEPC Interventional Cardiology Part of the ECHSA-CD*. Of note, this user interface is the same user interface that cardiac surgeons have used in ECHSA-CD for 22 years.
- Demographic data are comparable to the surgical dataset.
- A specific patient code for each patient will be created which anonymizes the data completely. Only this code is submitted to the server, while identifiable patient specifics remain locally stored.
- Table 1 documents the preliminary list of fields of data collection in the *AEPC Interventional Cardiology Part of the ECHSA-CD*.
- A User Manuel to the *AEPC Interventional Cardiology Part of the ECHSA-CD* will be published collaboratively by the AEPC Interventional Working Group in collaboration with the ECHSA Database Committee.
- Feedback reports will be developed collaboratively according to the needs of the AEPC Interventional Working Group and the ECHSA Database Committee.

**Table 1.** The Preliminary List of Fields of Data Collection in the *AEPC Interventional Cardiology Part of the ECHSA-CD*.

	Name	Type	Length	Mandatory	Comments
Patient					
	FirstName	Text	128	No	This field is mandatory in client software, exports to central database, and does not contain any personal data.
	LastName	Text	128	No	This field is mandatory in client software, exports to central database, and does not contain any personal data.
	LocalID	Text	128	Yes	
	Gender	Dictionary		Yes	(see table dictionaries)
	DateOfBirth	Date		Yes	
	DateOfDeath	Date		No	
	GestationalAge	Integer		No	weeks
	AntenatalDiagnosis	Yes/No		No	
	PrimaryOperationNo			Yes	(ID of patient's primary operation)
	DateOfLastFollowUp	Date		No	
	LastFollowUpNYHAClassification	Dictionary		No	
	PostRheumaticHeartDisease	Yes/No		No	
	DataVersion	Dictionary		Yes	(version of nomenclature used)
Diagnoses					
	Factor_code	Dictionary		Yes	(see table factors)
	Priority	Integer		Yes	
Admission					
	DateOfAdmission	Date		Yes	
	DateOfDischarge	Date		Yes	
	DischargeLocation	Dictionary		No	
	ReadmissionWithin30DaysReason	Dictionary		No	
					
					
CardIntervention					
	STSTerm - v3.4 - Diagnosis	Dictionary			
	Procedure STS term	Dictionary		Yes	
	Procedure performed	Dictionary		Yes	
	Procedure detail (drop down)	Dictionary		Yes	
	Manufacturer dropdown	Dictionary		Yes	
	Types of devices	Dictionary		Yes	
	Size	Integer (1-99)			millimeter
	Stents manufacturer	Dictionary			
	Type stent	Dictionary			
	Coils	Dictionary			
	Complication yes/no				
	Complication detail dropdown I	Dictionary			
	Complication detail dropdown II	Dictionary			
					
	Fluoroscopy time	Integer (1-999)			minutes
	Radiation dose				cGy*cm^2^
	Duration of procedure (skin in to skin out)	Integer (1-999)			minutes
	Success	Yes/No			

Abbreviations: AEPC, Association for European Paediatric and Congenital Cardiology; ECHSA-CD, European Congenital Heart Surgeons Association Congenital Database; STS, Society of Thoracic Surgeons.

Once the *AEPC Interventional Cardiology Part of the ECHSA-CD* is operational, all patients with pediatric and/or congenital heart disease at a participating institution undergoing cardiothoracic surgery and/or interventional cardiology will be entered into ECHSA-CD:

- Patients can be entered into the AEPC *Interventional Cardiology Part of the ECHSA-CD* even if they have never had cardiothoracic surgery.
- Patients can still be entered into ECHSA-CD even if they have never undergone an interventional cardiology procedure.
- Patients who have had both surgery and an interventional cardiology procedure will have data for both their surgical and interventional cardiology procedures entered into ECHSA-CD.

The initial data entry interface was checked by several interventional cardiologists for consistency and ease of data entry by the use of fictitious patients. The feedback from initial data entry was utilized to optimize data entry, reporting structure, and data verification.

**Figure 2.**
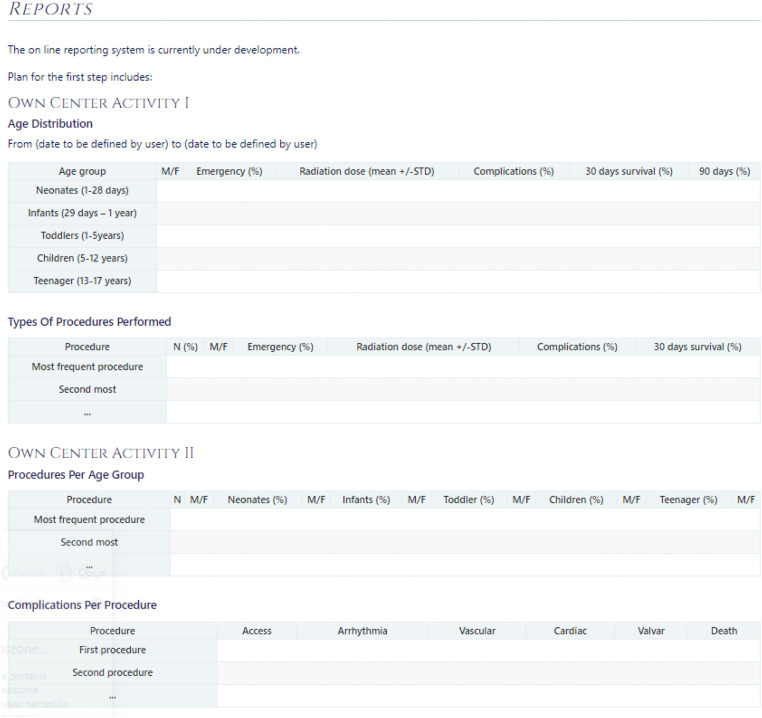
Example of feedback report to an individual center from the AEPC Interventional Cardiology Part of the ECHSA-CD. Abbreviations: F, female; M, male; STD, standard deviation.

A key feature of the new *AEPC Interventional Cardiology Part of the ECHSA-CD* is that own center reports can be obtained with one click, including the following information (Figure 2):

- Demographic data
- Interventions carried out in different age groups
- Types of interventions
- Radiation data
- Outcome data
- Follow-up data

This report should fit all quality demands which are required locally, regionally, and nationally. Furthermore, procedure-specific reports can benchmark own center results with national data (if more than three centers carry out the specified procedure) and European data. Also, own center results can be compared to all entered data regarding a given procedure on an international level. Importantly, comparison to verified data only will also be possible.

For international studies, the Council of AEPC has established a Steering Group, which will also be in dialogue with the ECHSA Database Committee and the ECHSA Research Committee. Thus, high-level quality data can be extracted and lead to high-impact publications.

The first version of the data entry software is already functional, and updates and corrections are in the process of being implemented. Based on feedback from users of the new *AEPC Interventional Cardiology Part of the ECHSA-CD*, the data entry module will be continuously refined, and the structure of the Feedback Reports will be continuously customized.

In December 2022, a contract was signed between ECHSA and AEPC that documented that AEPC and ECHSA agree to the following principles:

1. ECHSA and AEPC agree to develop the capability of ECHSA-CD to store and analyze data pertaining to pediatric and congenital cardiology catheter interventional procedures in the new “*AEPC Interventional Cardiology Part of the ECHSA congenital database.*” The governance, structure, and background of this collaborative initiative have been approved by AEPC and ECHSA and are detailed in this contract.
2. The *AEPC Interventional Cardiology Part of the ECHSA-CD* will provide:
  - appropriate lists and definitions of procedures,
  - relevant preintervention clinical, imaging, and/or pathophysiologic variables and related risk factors, as well as
  - procedure outcomes, including measures of technical success, complications, and possibly follow-up.
  - The same vocabulary/definitions for encoding diagnoses will be used for cardiology cases as already used for surgical cases.
3. *Within* ECHSA-CD, cardiology data will be treated in the same fashion as surgical data and can be accessed by contributing cardiology centers and analyzed by the same rules which apply to the surgical centers: Essentially, each cardiology center will have access to its own data and to cumulative anonymized data pertaining to the entire cardiology procedure dataset or to custom selected (“filtered”) subsets thereof.
4. ECHSA and AEPC will continue to cooperate to maintain and further develop the whole ECHSA-CD and the relevant data analytic tools.
5. The AEPC agrees to encourage its members to participate in the *AEPC Interventional Cardiology Part of the ECHSA-CD*.
6. AEPC will be acknowledged as an official ECHSA-CD partner on the ECHSA-CD website. Bidirectional links will be provided from the AEPC website to the ECHSA-CD website and vice versa.
7. The yearly fee per participating cardiology center will be the same as the fee for surgical centers, irrespective of the number of patients, admissions, or procedures entered.
8. If a center contributes both surgical and cardiology data, a 10% discount on the annual center participation fee will be applied to each department (Surgery and Cardiology).
9. Any publications resulting from the database utilizing interventional cardiology data will include recognition of both AEPC and the ECHSA-CD. Authorship involving interventional cardiology publications will be decided by AEPC. Publications involving surgical and interventional data will have balanced authorship of surgeons and interventional cardiologists.
10. This agreement will be valid for the duration of two (2) years, after which the agreement will be reviewed.
11. Each party shall have the right to terminate the agreement, with six months prior written notice to the other party.

Based on the formal agreement between AEPC and ECHSA that was signed on December 22, 2022, the new *AEPC Interventional Cardiology Part of the ECHSA-CD* is now operational, functional, and ready for the large-scale enrollment of patients. One can anticipate that this new part of ECHSA-CD will soon lead to important advances in pediatric and congenital cardiac care in the domains of patient care, research, and teaching, and that this new *AEPC Interventional Cardiology Part of the ECHSA-CD* will generate data that will be used to support:

- multiple presentations at national and international scientific meetings,
- numerous peer-reviewed scientific publications, and most importantly,
- feedback reports that allow benchmarking of individual programmatic to national and international aggregate data.

## Discussion

With the addition of the new *AEPC Interventional Cardiology Part of the ECHSA-CD* to the ECHSA-CD, ECHSA-CD has become the first multi-institutional, multinational database dedicated to pediatric and congenital cardiac care that seamlessly combines data from surgical operations and transcatheter interventional cardiology procedures; therefore, ECHSA-CD provides a previously unavailable platform to improve pediatric and congenital cardiac care across the world. The new *AEPC Interventional Cardiology Part of the ECHSA-CD* will allow centers to have access to robust outcome data from their own center, as well as robust aggregate outcome data for benchmarking. Each contributing center or department (cardiology or surgery) will have access to their own data, as well as aggregate data from the *AEPC Interventional Cardiology Part of the ECHSA-CD*. The new *AEPC Interventional Cardiology Part of the ECHSA-CD* will allow cardiology centers to have access to aggregate cardiology data, just as surgical centers already have access to aggregate surgical data. These data will help to improve the quality of patient care and identify risks related to certain techniques. The ECHSA-CD and the new *AEPC Interventional Cardiology Part of the ECHSA-CD* are tools for research activities and for the further development of the fields of congenital heart surgery and transcatheter interventions. National and international benchmarking will set the level of standard of care.

The strengths of ECHSA-CD and the new *AEPC Interventional Cardiology Part of the ECHSA-CD* include the following features:

- Use of a standardized international nomenclature (IPCCC),
- Use of an established database software platform,
- Use of established strategies for risk adjustment,
- Use of proven methods of data verification,
- Single access to both surgical and catheter interventional data,
- The large volume of data in ECHSA-CD, and
- The potential to track a single patient as this patient goes through various surgical and transcatheter interventional procedures during life.

Potential limitations and goals of the *AEPC Interventional Cardiology Part of the ECHSA-CD* include the following challenges:

- Strategies of risk stratification and risk adjustment for interventional cardiology procedures will need to be developed, standardized, and matured. Over the course of time, additional pre-procedural factors will likely be added to the *AEPC Interventional Cardiology Part of the ECHSA-CD* in order to facilitate the development of tools for risk stratification and risk modeling.
- Strategies of data entry for hybrid procedures will need to be developed (eg, surgical pulmonary valve replacement and distal pulmonary arterial stent insertion, and hybrid palliation of hypoplastic left heart syndrome).
- Strategies of risk stratification and risk adjustment for hybrid procedures will need to be developed (eg, surgical pulmonary valve replacement and distal pulmonary arterial stent insertion, and hybrid palliation of hypoplastic left heart syndrome).
- Strategies will need to be developed to determine the primary interventional cardiology procedures if more than one interventional cardiology procedure is performed during the same intervention (eg, combined atrial septal defect device closure and pulmonary arterial balloon dilation or stent insertion, or other combinations of transcatheter procedures).
- Both ECHSA-CD and the *AEPC Interventional Cardiology Part of the ECHSA-CD* do not currently serve as platforms for longitudinal follow-up. A future goal of both ECHSA-CD and the *AEPC Interventional Cardiology Part of the ECHSA-CD* is to include a longer-term follow-up module. It is an absolute fact that of all the information that we currently lack, consistent, and structured follow-up data is at the top of the list.

## Conclusion

The new *AEPC Interventional Cardiology Part of the ECHSA-CD* will allow centers to have access to robust surgical and transcatheter outcome data from their own center, as well as robust aggregate outcome data for benchmarking. Comparison of surgical and catheter interventional outcomes will strengthen decision processes. A study of the wealth of information collected in the database will also contribute toward improved early and late survival, as well as enhanced quality of life of patients with congenital heart disease treated with surgery and interventional cardiac catheterization across Europe and the world. In the final analysis, the addition of the new *AEPC Interventional Cardiology Part of the ECHSA-CD* to the ECHSA-CD transforms ECHSA-CD into the first multi-institutional, multinational database dedicated to pediatric and congenital cardiac care that seamlessly combines data from surgical operations and transcatheter interventional cardiology procedures; therefore, ECHSA-CD provides a previously unavailable platform to improve pediatric and congenital cardiac care across the world.

## Supplemental Material

sj-docx-1-pch-10.1177_21501351231168829 - Supplemental material for Combining Congenital Heart Surgical and Interventional Cardiology Outcome Data in a Single Database: The Development of a Patient-Centered Collaboration of the European Congenital Heart Surgeons Association (ECHSA) and the Association for European Paediatric and Congenital Cardiology (AEPC) Click here for additional data file. Supplemental material, sj-docx-1-pch-10.1177_21501351231168829 for Combining Congenital Heart Surgical and Interventional Cardiology Outcome Data in a Single Database: The Development of a Patient-Centered Collaboration of the European Congenital Heart Surgeons Association (ECHSA) and the Association for European Paediatric and Congenital Cardiology (AEPC) by Jeffrey P Jacobs, Thomas Krasemann, Claudia Herbst, Zdzislaw Tobota, Bohdan Maruszewski, Jose Fragata, Tjark Ebels, Vladimiro L Vida, Ilkka Mattila, Andrzej Kansy, Boulos Asfour, Jürgen Hörer, Attilio A Lotto, M Sertaç Çiçek, Petru Liuba, Sven Dittrich, Massimo Chessa, Regina Bökenkamp, Gurleen Sharland, Katarina Hanséus, Nico A Blom and George E Sarris in World Journal for Pediatric and Congenital Heart Surgery

## Abbreviations

AEPC: Association for European Paediatric and Congenital Cardiology
CD: Congenital Database
CHD: congenital heart disease
CHSD: Congenital Heart Surgery Database
EACTS: European Association for Cardio-Thoracic Surgery
ECHSA: European Congenital Heart Surgeons Association
IPCCC: International Pediatric and Congenital Cardiac Code
STS: Society of Thoracic Surgeons
