# Supplementary material for: Combining Congenital Heart Surgical and Interventional Cardiology Outcome Data in a Single Database: The Development of a Patient-Centered Collaboration of the European Congenital Heart Surgeons Association (ECHSA) and the Association for European Paediatric and Congenital Cardiology (AEPC)
Source: World J Pediatr Congenit Heart Surg. 2023 Jul 6;14(4):464–73. doi: 10.1177/21501351231168829 (PMC10411030; doi:10.1177/21501351231168829)
Supplement: sj-docx-1-pch-10.1177_21501351231168829 - Supplemental material for Combining Congenital Heart Surgical and Interventional Cardiology Outcome Data in a Single Database: The Development of a Patient-Centered Collaboration of the European Congenital Heart Surgeons Association (ECHSA) and the Associati [file sj-docx-1-pch-10.1177_21501351231168829.docx]

**Appendix. Quick Users’ Guide** demonstrating multiple screen captures of the user-friendly data entry interface that was developed for the ***AEPC Interventional Cardiology Part of the ECHSA-CD***.

Of note, this user interface is the same user interface that cardiac surgeons have used in ECHSA-CD for 22 years


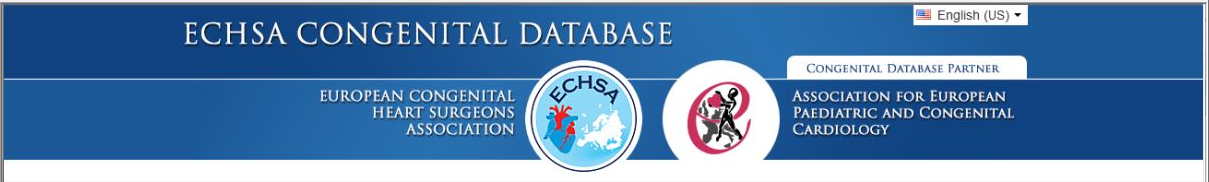


**Quick User’s guide**

January 2022

1. Allow windows to run the database. Antivirus programs like AVG may need to check the files. Take your time. Prepare a coffee or tea ...

2. Use the "Default" settings. The database will launch.


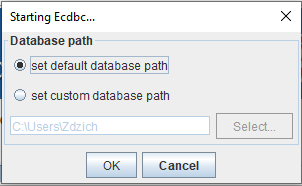


3. Put in a 3-letter hospital code in CAPITALS, which you will receive via e-mail after registration on the website. (Once the database is fully live, this code will be provided by the AEPC-ECHSA team). Enter the remaining details. Click "save" and close this window.


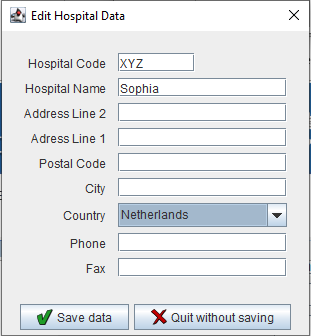


4. Go to data-preferences: Set preferences:

- Default operation type: interventional cardiology.
- Date format (YYYY-mm-dd or dd-mm-yyyy)., and
- Click the default under "confirmations".


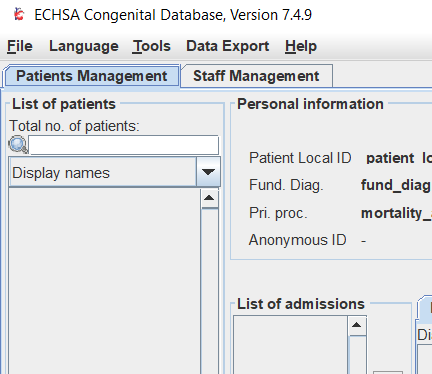


5. Select your language or leave English.

6. Under "help" you can find an online manual, but a very short introduction here is provided here.

7. Go to "**Staff Management**"

- Add new person:
- Enter all interventionalists.


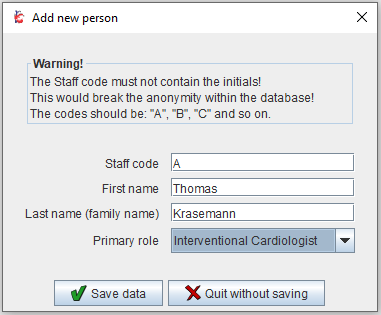


8. Go to Patient Management - and now it seriously begins...


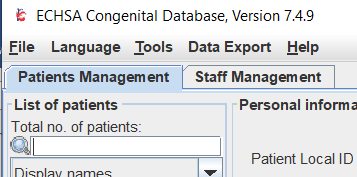


Please use the following order to enter patient data.

The rationale for this approach is explained in the text below.

1. Click "add patient".


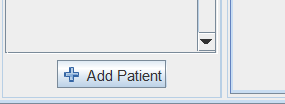


2. Enter First and Last Name, Date of birth, Hospital Number, Gender, and the Place of birth (“Date of birth” and “Place of birth” are important to identify patients even if they changed Last Names or moved to another center, etc.). These data will be transformed into a unique identification code, which is later transferred to the server. No patient identifiable data will be transferred. Save data.


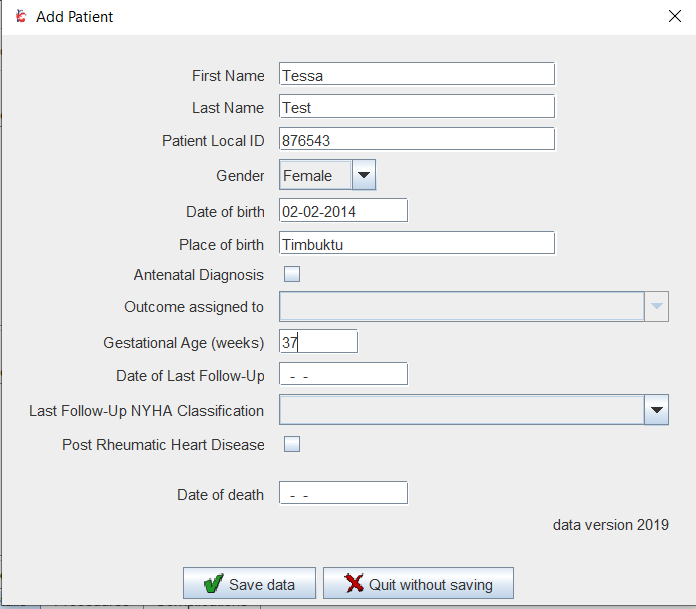


2. Add diagnosis using the "+" button. You can either search alphabetically or using one of the diagnosis groups and then the dropdown. It is important to add all diagnoses, as interventions are linked to the diagnosis. (i.e., you are not able to enter closure of a duct if the diagnosis is ASD). These diagnosis groups are based on surgical groupings; therefore, one can choose to use or ignore the diagnosis groups while entering the diagnoses. One should add all diagnoses the child has (e.g., ASD and VSD and PDA).
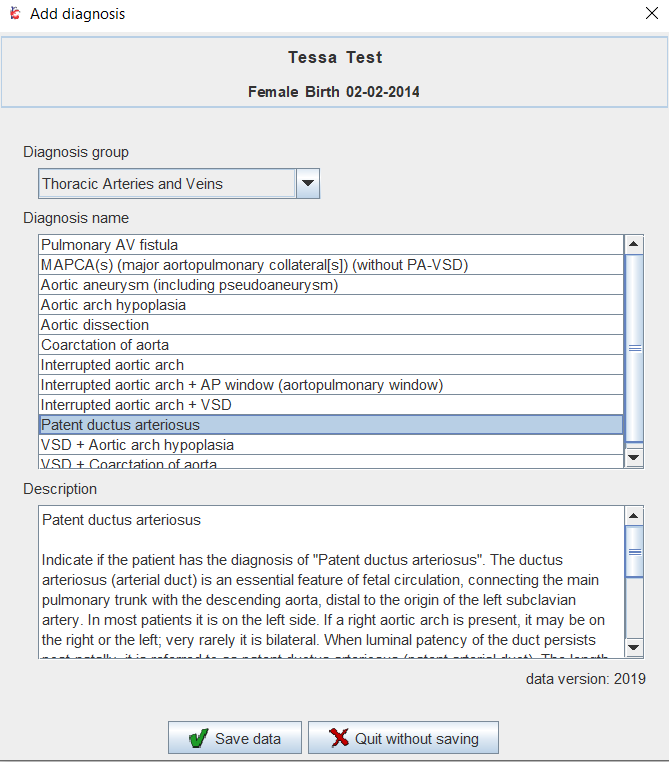


3. Add an admission using the "+" button. Click on the "+" at the list of admissions field. (No intervention can be carried out without an admission). Enter admission date.


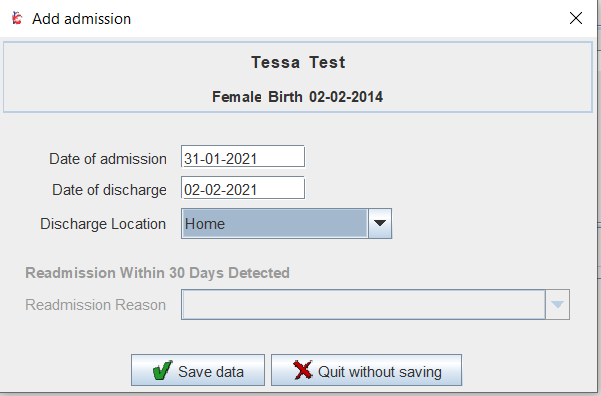


4. Add an intervention using the "+" button. Click on the "+" at the list of interventions.


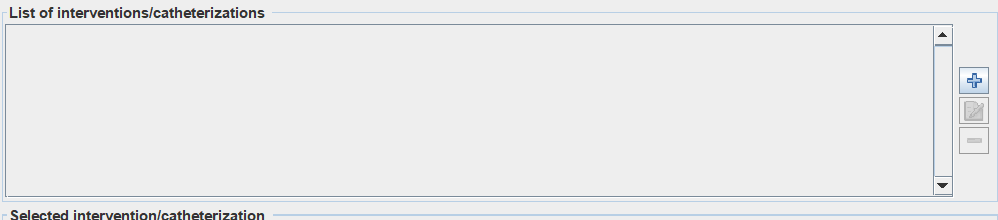


5. A new window opens.

- Fill in date of intervention (needs to be within the admission period).
- Enter “Yes” or “No” for Success. (Success is defined as having achieved the goals set for the intervention. [In the case of a diagnostic catheterization, success is defined as obtaining all data acquired as planned]).
- The field named “Treatment performed by” opens a dropdown of the staff entered previously. Two operators are standard.
- Fill in demographic data.
- Fill in fluoroscopy data (not mandatory, but important for later reports).
- Click access sites.
- Complications: Enter “Yes” or “No”.
- If you know the 30 and 90 days status, please fill in. (This can be done later, and you will be reminded to fill in after this timeframe automatically when opening the database again).
- Save data.


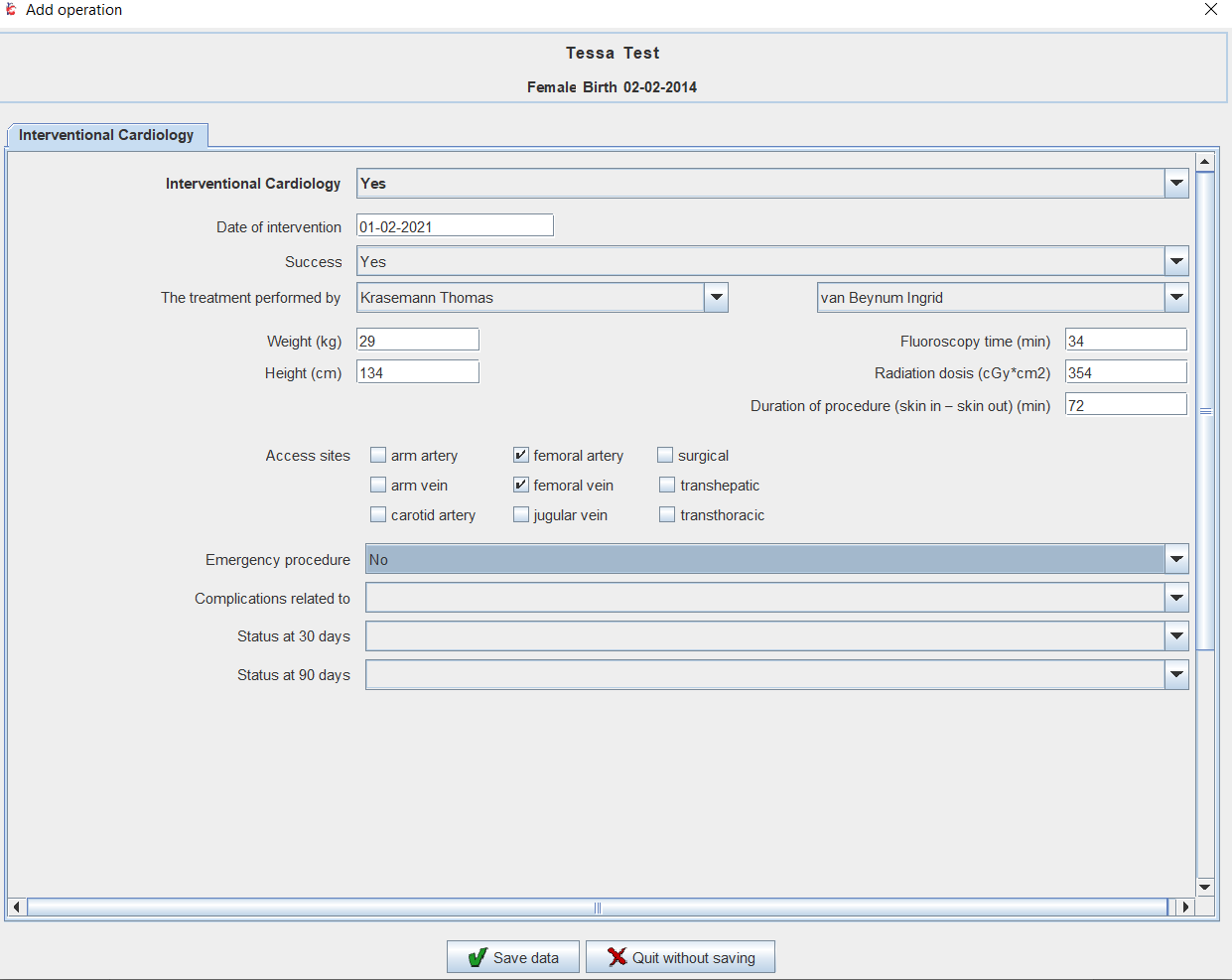


6. Click once on the intervention you just prepared. It will highlight.


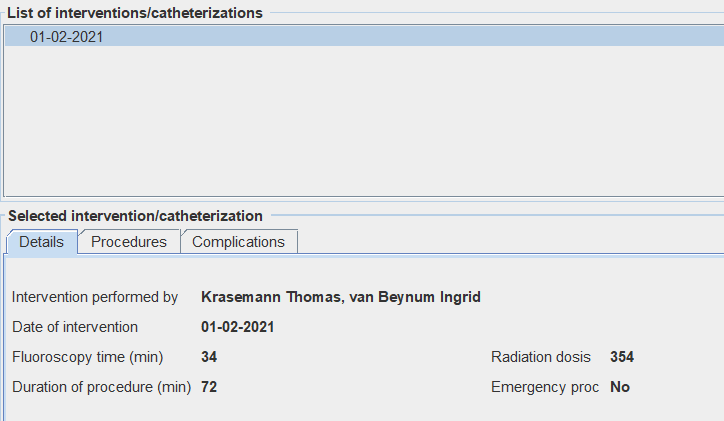


Now click on the register "procedures" and then on "+". Now you are able to add specific procedures related to the previously entered diagnosis.


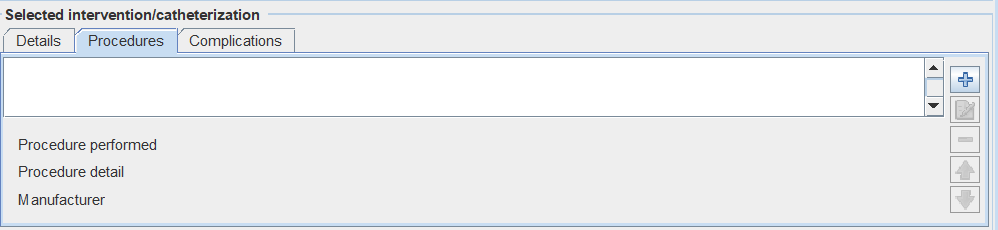


- First choose the Diagnosis from the dropdown.
- Then choose the Procedure (Intervention) performed.
- Please also choose the STS code - this is for comparison with other databases at a later stage. Not mandatory, but useful. There might not be an adequate STS code for every procedure- leave blank if uncertain.
- Procedure detail allows to choose the device type.
- Manufacturer opens a dropdown of manufacturers.
- Enter device size (The device size is defined as the diameter for a stent or device with a waist; and otherwise, the device size is defined as described by the manufacturer).
- Device specification can either be the serial number or some specifics regarding position- this field is free text.
- Save data. The window closes.


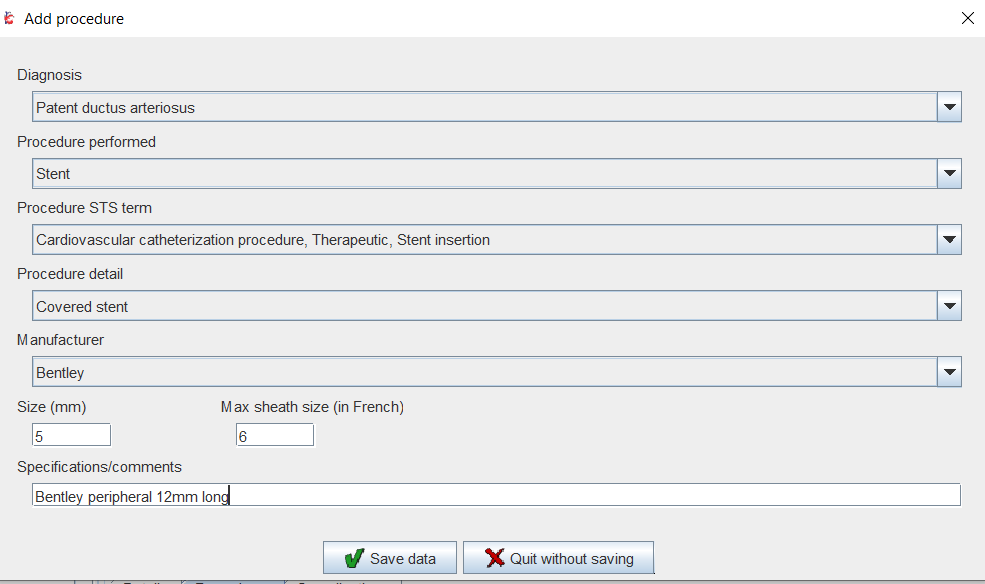


Most of the above is not mandatory, but the more data one enters, the more one might ultimately get out of the database.

If more than one intervention is performed (e.g., VSD closure and PDA closure), then add another Procedure by using the "+" and enter the data accordingly.

After the window is closed, the previous screen returns.

If a complications occurred, click on the Complications tab and use the "+" to enter a complication.


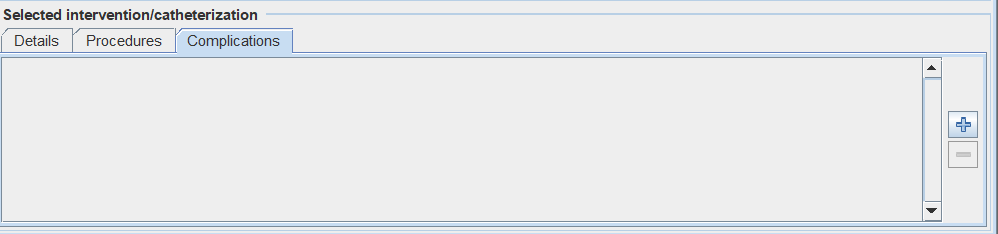


The software provides a dropdown menu with complication groups. It is recommended to use this dropdown menu.


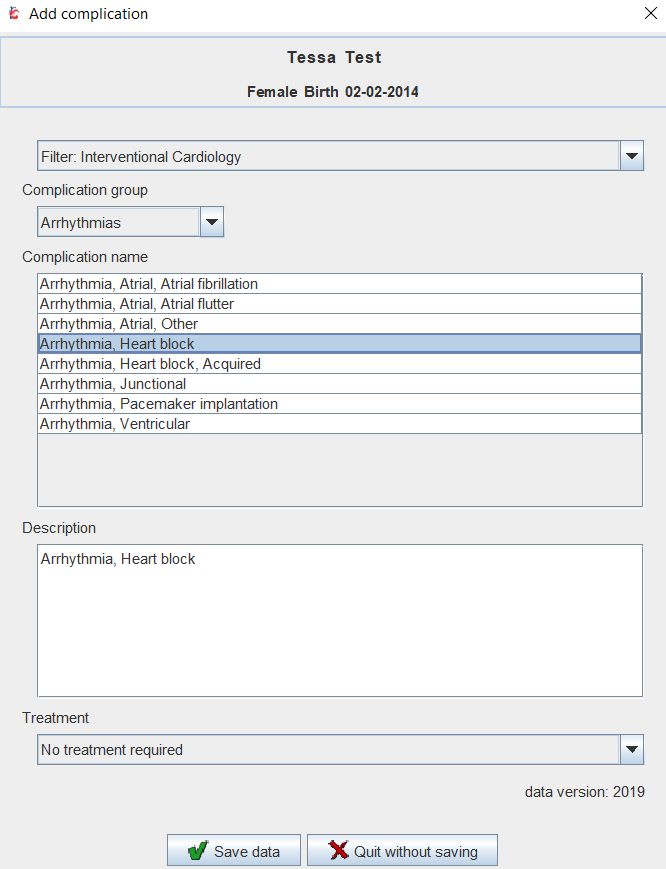


Finally, go to "Data Export" and save the data on disk.

Then, data entry for this procedure is complete.

If more than one diagnosis is present, or more than one procedure (intervention) is performed on the same lesion (e.g., ballooning and stenting of a pulmonary artery), then please enter both diagnoses and/or both procedures.

Similarly, if a patient has more than one admission with interventions, please start again with adding the additional admission(s), and then add the associated diagnosis(es), and then add the associated procedure(s).
